# Geriatric oncology: problems with under-treatment within this population

**DOI:** 10.7497/j.issn.2095-3941.2015.0081

**Published:** 2015-12

**Authors:** Divya Swaminathan, Vikram Swaminathan

**Affiliations:** ^1^Norwich Medical School, University of East Anglia, Norwich NR4 7TJ, UK; ^2^Health Education North West, Liverpool L3 4BL, UK

**Keywords:** Cancer, elderly, management, geriatric oncology

## Abstract

We are currently faced with an aging population, which is rapidly growing worldwide. Two thirds of cancer occurs in the over 65-year age group. Societal conceptions from the past have created ageist stereotypes; old age is associated with frailty and the elderly are perceived to be destined for deterioration and loss of independence. Cancer within the elderly is also subject to these stereotypes, with elderly cancer patients considered by some not as likely to recover as younger patients with cancer. We summarise and review the current concerns regarding elderly management and treatments utilised for the management of oncological disease in the elderly, and discuss the impact of under-treatment within this population.

## Introduction

The expressions elderly and geriatric are used widely in modern medicine and are defined as terms describing the old or aged person^1^. However, when asked to specifically define old age, there is no universally accepted interpretation in place^2^. The World Health Organisation (WHO) proposes that most countries define elderly as being over the age of 65 years, generally recognised as retirement age^3^. In our society, this demographic is unfortunately susceptible to misconceptions and ageist stereotypes, creating complexities in the medical management of the eldly.

We are currently faced with an aging population, which is rapidly growing worldwide. Healthy life expectancy is increasing and people are living for longer periods of time^4^. In 2010 the number of over 65’s was estimated to be around 534 million, the equivalent of 8% of the worlds’ population^5^. By 2021, this is predicted to rise to approximately 10%^6^. Moreover, by 2050, this is anticipated to almost triple in number to 1.5 billion; 16% of the world’s population will be of retirement age^2^. As a result of these demographic changes, it could be argued that our accepted definition of elderly and the associated age of 65 years is no longer medically relevant and it should be changed to an age of at least 75 years and over; perhaps this cut off would provide greater meaning. Nonetheless in the current era of geriatric medicine, age alone is no longer enough to characterise an individual^6^.

Age is an indirect risk factor for cancer; extended life duration is coupled with increased exposure to carcinogenic factors alongside greater time for the accumulation of genetic changes, which could eventually result in the generation of a tumor. Two thirds of cancer occurs in the over 65-year age group, a noteworthy proportion of individuals^6^. Statistics collected between 2009 and 2011 by Cancer Research UK present the male to female ratio of diagnosis as 1.06:1 per year (60,828:57,221) within the elderly aged over 75 years^7^^,^^8^. About 70% of cancer related deaths occur within this population^9^. The prevalence of certain cancers varies with gender. In order of decreasing incidence, prostate, lung, and bowel cancers contribute to 57% of cancers diagnosed in elderly males. Females are most commonly affected by breast cancer (21%), followed by lung, bowel, stomach, and uterine cancer^10^.

Societal conceptions from the past have created ageist stereotypes; old age is associated with frailty and the elderly are perceived to be destined for deterioration and loss of independence^11^. Cancer within the elderly is also subject to these stereotypes, with elderly cancer patients considered by some not as likely to recover as younger patients with cancer. However, this is not necessarily the case and currently elderly members of the community are often able to work and play an active role in society^12^. Ageism is defined as ‘an attitude that discriminates, separates, stigmatises or otherwise disadvantages older adults on the basis of chronological age’^13^. In healthcare, it alludes to the idea that considerable deterioration is always a normal part of aging and that older individuals may not benefit as much as a younger individual from certain treatment, and therefore should perhaps not be offered it. Under-treatment in the geriatric patient is reinforced by the ageist approach to care and although this approach is built on the basis of avoiding harm, it does not follow the principles of consent, capacity and acting in a patient’s best interest^14^. We summarise and review the current concerns regarding elderly management and treatments utilised for the management of oncological disease in the elderly, and discuss the impact of under-treatment within this population.

## Issues associated with the management of the elderly

The management of geriatric patients does not come without challenges. The aging process is accompanied by multisystem physiological changes in the body, including deterioration of vision and hearing, reduced kidney function, reduced gastro-intestinal motility, reduced efficiency of the cardiovascular and respiratory systems, decreased bone density, and reduced mobility to mention a few^15^. Although these changes are natural they render the elderly less able to manage, with a reduced reserve for stresses placed on the body and more susceptible to pharmacokinetic and pharmacodynamics interactions, which may lead to toxicity^2^.

Any psychological or cognitive decline can alter the amount of patient support required, which may be accompanied by difficult social or financial circumstances^16^. Limited social support and difficulty accessing transport and healthcare create further hurdles in the pathway to obtaining treatment for geriatric patients. All of these factors may contribute to the effectiveness of treatment and a patients’ prognosis. Evidence suggests that good family support networks increase patient tolerance towards more extensive treatment regimens^1^. It is clear that even elderly patients of good health still have different health needs compared to younger patients^8^.

## Financial burden of cancer care in the elderly

The cost of treatments can vary globally, depending on the intervention, the site, and the stage (severity) of cancer being treated. If treatment costs continue to rise annually by 2%; breast and prostate cancers will produce the largest increases in expense, 32% and 42% relatively, simply due to the fact that these cancers will remain most prevalent in the population^2^.

According to Macmillan Cancer Support, the financial impact of cancer on an individual is most considerably influenced by age, the stage of their cancer, and their social class (employment and income)^17^. In addition to treatment costs, these patients must be prepared to fund transportation to and from appointments and perhaps to specialist centres. Cancer may also result in increased costs for the family of the patient; addition funding is required to support the elderly patient, due to declining ability for the patient to manage their required activities of daily living.

A report published by the department of health in 2014 presented figures regarding emergency hospital admissions. The years 2012/2013 saw 82,600 admissions due to cancer, representing 3.7% of all emergency admissions. Geriatric patients may sometimes be referred to as ‘hazards of hospitalisation’, simply due to their greater risk of hospital related complications. These include malnutrition, pressure ulcers, falls, delirium and hospital acquired infection^18^. Emergency admissions to hospital can be expensive and elderly patients may have a higher occurrence of re-admission. Prevention of this is therefore a collective objective in the health service.

With a growing bank of people within the elderly demographic and an inevitable rise in the prevalence of cancer, we need to work towards providing optimal treatment in a cost effective manner. With limited resources and funding, there is a need to provide this optimal care to everyone, including the geriatric population.

## Management of geriatric oncology patients

There is plenty of evidence to suggest that under-treatment of the elderly is a very real issue. This under-treatment arises for many reasons. Firstly, the lack of elderly patients in clinical trials has led to insufficient evidence and gaps in our knowledge as to what are the most effective treatments for geriatric oncology patients. This in turn results in a lack of guidelines to support evidence-based decisions, and so patients may not receive the most effective treatment and are undertreated. In addition, iatrogenic factors play a role. The ageist approach to care of geriatric patients gives rise to bias and misconceptions as to what is best for the patient and which treatments they can tolerate. Lower risk and less aggressive interventions may appear a more attractive option to physicians, in order to avoid patient morbidity or mortality. Nonetheless, 15,000 older cancer patients die prematurely due to this approach and under-treatment leads to a reduced 5-year patient survival^1^^,^^2^.

A retrospective evaluation reported data involving 212 patients over 80 years old with newly diagnosed breast cancer at the University of Texas MD Anderson Cancer Centre between 1989 and 2004. Those treated only with primary endocrine therapy in comparison to endocrine therapy in combination with surgery had a significant reduced survival (*P*=0.001). The study also illustrated that 57% of these patients with a huge proportion, were under-treated when consulting the existing guidelines^19^. The conclusions from this study held great importance. It not only highlighted the benefits of multidisciplinary therapy in the elderly, but also emphasised that under-treatment leads to reduced cancer survival and impaired prognosis^1^.

Diffuse large B-cell lymphoma is a subtype of non-Hodgkin’s lymphoma and a malignancy that is increasing in prevalence throughout the elderly population. It is manageable and potentially curable, like several other cancers. In concordance with the reasons already mentioned, geriatric patients are largely under-represented in clinical trials and so treatment decisions for this cancer is generally based on evidence from younger patients. As a result some patients are given sub-optimal therapy in an attempt to avoid toxicity, perhaps having negative implications on prognosis. Alternative regimens may be considered for geriatric patients which would be deemed as under-treatment in younger populations. The issue of geriatric under-treatment is therefore very apparent. The international society of geriatric oncology (SIOG) recently chose to review the treatment of these patients and outline more effective guidelines for management to target this potentially detrimental care^20^.

There are an increasing number of treatments and potential options now available for the medical and surgical oncological management of the geriatric patient. Current treatment pathways available include surgery, radiotherapy, chemotherapy, biological therapies, pharmacological treatment and endocrine therapy. There are a handful of further treatments also available that are not regarded as frontline. All of these treatments may be used individually as monotherapy, or in collaboration with adjuvant supportive treatment.

### Surgical

According to the Royal College of Surgeons England, there were 4.7 million surgical admissions in 2013-2014^21^. The most common procedures performed were hernia repairs (120,198) followed by hip replacements (115,758) and knee replacements (81,590). Despite the prevalence of cancer within the population, cancer related surgery did not appear amongst the top 10 surgeries performed.

Surgery is perhaps the branch of treatment that causes most debate. It is perhaps misconceived by some health professionals and certainly by the public that old age is contra-indication to oncological surgery. Surgery is invasive and inevitably there are greater risks associated, including lengthy hospital stays, ICU admissions and undesirable complications such as premature mortality. Despite this, surgery in an older patient can provide similar success with cancer treatment as in younger patients with well-regarded evidence to support the feasibility of surgery within this age group^1^^,^^22^. It is important to remember that surgical therapy can be curative; surgery is reported as ‘the most effective cancer-ablative therapy’^22^. Surgical resection of cancer can reduce costs by removing the need for ongoing long-term treatments to manage the cancer in elderly patients.

Surgical interventions come in several forms, ranging from simple local excision to the removal of entire glands such as in a mastectomy^23^. As patients remain healthier for longer, the breadth of patients within the surgical group is increasing. Lung cancer is characterised by an association with age^22^. Over 65% of patients with lung cancer are over the age of 65 years when they are initially diagnosed. Furthermore, 25% of patients diagnosed are 75 years or older with 30% of patients who die from lung cancer within this latter demographic. A lung lobectomy is the current standard procedure for patients of any age, and carries an operative mortality risk of 1.4%^22^. Results from randomised trials suggest that there is no increased mortality risk related to this surgery with proceeding age^24^. Further research is required to identify peri-operative mortality rates that are representative for the elderly population but these figures suggest that the elderly patient should not be excluded from surgical interventions on the basis of age alone.

Between the years 1989 and 1999, Bouchardy *et al.*^25^ performed a study reviewing 407 breast cancer patients over 80. The study demonstrated that under-treatment of the elderly was a very real issue and that treatment provided was independently associated with age. Of all the participants, 12% of women had no treatment, 33% had a mastectomy, 32% received hormone therapy in the form of tamoxifen, 14% had breast conserving surgery with adjuvant therapy and 7% had breast conserving surgery alone^25^. The primary outcome (5-year specific breast cancer survival) produced notable findings. Five-year survival figures were presented as 46% for women with no treatment and 51% for those given tamoxifen. Contrasted with an 82% 5-year survival from the mastectomy patients and a 90% survival from those who had breast conserving surgery and adjuvant treatment, not only do these results suggest that under-treatment leads to reduced survival and worse prognosis, they highlight the benefits of surgical intervention for geriatric patients.

Nevertheless although surgery should be considered optimistically, it remains a high-risk intervention. The overall peri-operative mortality rate stands at 1.2% for the general population within 30 days of surgery and this rate substantially rises with increasing age^26^^-^^29^. The risk of death rises significantly to 19.8% with major surgery in the over 90s age group^30^. It is important to remember that proposing surgery to prolong life still carries a considerable risk (**Table 1**).

**Table 1:** Mortality rates during surgery split by age groups (34-38)

Age (years)	Risk of peri-operative mortality (%)
<59	1.2
60-69	2.2
70-79	2.9
80-89	5.8-6.2
≥90	8.4

The mortality associated with surgical intervention is not only associated with age but with the nature of the surgery itself. The risk associated with aggressive, invasive surgery such as pancreatic or oesogastric surgery is not comparable to the risks associated with breast or lung cancer surgery. However, a study driven by the current changing demographics and elderly population reviewed 438 patients over a 9-year period following a pancreatic resection. The study concluded that elderly patients can safely endure invasive pancreatic surgery and that age alone should not exclude them from this group. Nevertheless there was still significant risk of morbidity, poor prognosis and reduced quality of life as a result^31^.

A further concern is the issue of screening for cancer in the elderly population. Perhaps the importance of early diagnosis and prevention is overlooked and not stressed sufficiently within the geriatric population. Earlier diagnosis could reduce the need to high risk emergency surgery in this population.

In an era where cancer is becoming increasingly by prevalent and diverse, there is no longer room for a ‘one criterion fits all’ parameter in the form of an age threshold. The surgical approach should arguably never be excluded despite patients’ age. Patients can be individually assessed and evaluated for their fitness for surgery using various tools. Using a more holistic approach and considering a combination of factors (including health assessment), many elderly individuals may be deemed fit for surgical treatment of their cancer. Fear associated with surgical outcomes in addition to a lack of guidelines leads to surgical options being inadequately considered, resulting in under-treatment of these geriatric cancer patients.

### Medical

The UK budget for the cancer drug fund was raised from 200 million pounds in 2013/2014 to an estimated 340 million pounds in April 2015^32^. However, earlier in 2015 several cancer drugs were removed from The National Health Service (NHS) funding simply due to their expense. Examples include lapatinib, eribulin, and everolimus, all used in the treatment of breast cancer; pemetrexed, which is used to treat lung cancer, and cabazitaxel, used for late-stage hormone resistant prostate cancer. These cut backs are estimated to affect almost 8,000 patients in the UK, many of them with advanced disease and perhaps likely to be geriatric^33^.

With advancements in the medical management of cancer, recent years have seen a decline in the incidence of mortality due to breast cancer in younger women, however, this trend is not been observed in older women^1^. Advancements in pharmaceutical treatments created by the drug industry drive the development of more effective medicinal therapies for use in oncology. However, this progression in efficacy is inescapably coupled with increasing expense and financial repercussion. An example is traztuzumab (Herceptin) which is used in the treatment of breast cancer. A course of traztuzumab costs on average approximately 25,000 pounds with the cost effectiveness estimated at 400,000 pounds per recurrence prevented^6^. We will become ever more reliant on these drugs in the future but unsurprisingly many patients may not be able to afford to fund these treatments privately.

Evidence suggests that endocrine therapy should not be used in geriatric patients at all, particularly with those that are frail or those with tumors that are at low risk^34^. This is because these hormonal therapies may cause several unfavourable side effects such as deep vein thrombosis; bone loss and musculoskeletal conditions. These negative effects largely outweigh the potential therapeutic benefit in elderly patients. However, as with all treatments there are exceptions to the rule and the decision must be made on an individual basis. Medical management of oncology in the elderly should not be dismissed purely on an impression of ability to tolerate side effects, as this can lead to under-treatment within this population. For example, with the treatment of breast cancer, hormonal therapy can be used in both young and older patients; it remains the adjuvant therapy most readily used in older women due to the predominance of cases with estrogen receptor (ER) expression. Tamoxifen would perhaps be recommended for use in geriatric care. Tamoxifen also helps to prevent reduced bone density, a physiological change associated with old age. The use of any drug in the geriatric patient requires close monitoring^35^.

### Chemotherapy

The cost of chemotherapy or radiotherapy per episode, which may be comprised of 3-4 sessions, can be as overwhelming as 35,000 pounds^36^. Unsurprisingly, the use of chemotherapy in geriatric oncology has the smallest evidence base. The potential toxicity of treatment also can make clinical trials unethical for the geriatric population. The narrow therapeutic index of chemotherapeutic drugs makes dose selection particularly complex. An additional physiological change associated with aging is reduced function of the CYP2 enzyme^9^. This biological catalyst plays a key role in the metabolic pathway involved in the action of chemotherapeutic drugs, important to minimising toxicity. The current lack of evidence creates a barrier in establishing the effectiveness of this treatment in older patients. Current practice suggests that adjuvant chemotherapy is indicated only after careful consideration of a combination of factors, such as recurrence risk and concurrent mortality risk^26^. In one study, 318 patients ranging from age 80 to 92 years were reviewed between 2005 and 2010. All participants were receiving chemotherapy for solid malignant tumors and the consequences of this treatment were investigated. The primary outcome of the study was discontinuation of chemotherapy due to toxicity and there were several secondary outcomes regarding adverse effects. Chemotherapy was first line for 89% of participants; 41% ordered an upfront dose reduction, 32% withdrew from treatment due to the toxicity and notably 32% of patients were hospitalised^27^. Inspite of a small study, this evidence quite clearly implies that chemotherapy in older patients poses quite serious risks and the requirement for effective methods of patient selection are emphasised. Clinicians must question whether the risks presented are worthwhile for the therapeutic benefit achieved from chemotherapy treatment of oncology in geriatric patients.

Several randomised trials have been published regarding metastatic colorectal cancer to investigate reduced-dose chemotherapy options and assess predictors of outcome in frail patients with advanced colorectal cancer. A study published in 2011 once more highlighted that although often treated with chemotherapy, the elderly are under-represented in such clinical trials. FOCUC2 aimed to investigate reduced-dose chemotherapy options and assess outcomes in frail patients with advanced colorectal cancer^37^. Similarly further trials include FFCD 2001-02, AVEX and PRODIGE 20. Trials are ongoing and therefore display that progress is being made to improve the evidence base for oncological treatment of geriatric patients.

In addition to this, a promising instrument developed to predict chemotherapy toxicity by the Cancer and Ageing research group has been recently trialled and supported in several clinical environments including geriatric oncology. Also, the first prospective clinical trial regarding elderly women and ovarian cancer is being supported by the Elderly Taskforce of the Gynaecological Oncology group. The American Society of Clinical Oncology (ASCO) plays an active role in geriatric oncology, introducing a geriatric oncology issue exploration team, establishing further educational materials, creating the B.J Kennedy Award for Excellence in Geriatric Oncology and establishing a geriatric oncology element to the Cancer Education Committee. All these interventions aim to provide evidence based guidance for the management of oncology in the geriatric population, aiming to improve the future of geriatric oncology^29^.

### Radiotherapy

Evidence suggests than any benefits of radiotherapy appear to decrease as age increases^1^. Radiotherapy can be very debilitating, particularly to older patients, due to the effect on the entire body, as well as complications locally where applied. Radiotherapy treatment can be an ongoing commitment, requiring multiple sessions over a long period of time. This can be physically taxing, and is accompanied by the financial burden of retreatments. This may lead to a lack of compliance or withdrawal from therapy, as patients cannot tolerate the treatment. A recent publication in the European Geriatric Medicine journal re-evaluated some retrospective data regarding the treatment of breast cancer patients over 65 years of age. The authors investigated 79 patients who had been treated and followed-up in the Cumburiyet University Medical Oncology Department and found that 66.6% of patients receiving chemotherapy and/or radiotherapy had related side effects^38^. However, the study concluded that neither therapy was more toxic in elderly patients compared to younger patients, therefore treatment of each age group should be equivalent^38^.

## Appropriate health assessment and treatment for geriatric oncology patients

The treatment of geriatric patients may be complicated by a spectrum of physical, psychological and social challenges. The factor of age alone cannot be just justified as the reason for treatment selection or dismissal. As with any age group, each patient is different and must be individually evaluated to establish the most effective management. Within the general population, standard methods of medical evaluation are effective but are not sufficient for assessment of elderly patients, as they do not account for factors such as cognitive decline, reduced mobility or iatrogenic disorders^1^.

Acknowledgement of the importance of the initial evaluation in the medical community has led to the creation of several tools for bespoke assessment, which play a key clinical role. A useful example is the comprehensive geriatric assessment (CGA). The use of this evaluation enables treatment selection acting in the patient’s best interest as it removes any ageist bias. This standardised tool reviews several domains (**Table 2**)^39^.

**Table 2:** Domains reviewed in CGA

Medical	Functional	Mental health	Social	Environmental
Co-morbidities	Gait & balance	Cognition	Informal support	Use of telehealth technology
Disease severity	Exercise status	Mood	Social network	Transport
Medication review	Activities of daily life (ADLs)	Anxiety	Family, friends & visitors	Home comfort & safety
Nutritional status	Instrumental ADLs	Fears	Daytime activities	Occupational health
Other problems			Eligibility for care resources	Accessibility to local resources and facilities

CGA, comprehensive geriatric assessment.

A recent study evaluating the use of the CGA on patients after hospital admission came to several statistically significant conclusions^40^. The meta-analysis found several benefits of performing a CGA including a reduction in patient deterioration and a decreased incidence of death (*P*=0.001). Evidence was also presented showing that elderly patients were more likely to survive hospital admission, return home, and remain alive for the return home and remain alive for the following year if a CGA was performed during their inpatient stay. This research clearly highlights the effectiveness of geriatric assessment. In addition to these advantages an overall benefit in cognitive measures was also shown (*P*=0.02)^40^. These conclusions were reinforced by another study, which also presented benefits of an inpatient CGA *vs.* no CGA in 10,427 participants from a varied elderly population^41^. Twenty randomised control trials were included together in a systematic review. In addition to the benefits expressed in the previous study, this review concluded that ‘for every 100 patients receiving a CGA, three more will be alive in their own homes’^41^. It is interesting how the use of such a simple methodological tool can make such a difference to quality of life. Additional recently adopted and regularly used tools include the mini-geriatric and G8 score. There is a wealth of evidence to suggest that these methods of evaluation are particularly effective. A study evaluating the G8 and fTRST screening tools in geriatric care concluded that both measures were effective for assessing patients and determining their prognosis with regards to functional decline and overall survival. A total of 937 patients aged over 70 years were included. These individuals had a malignant tumor and a new cancer event requiring a treatment decision^42^.

Similar positive results were shown by a small study carried out involving elderly patients with different digestive cancers. The study aimed to assess the feasibility of mini geriatric assessment (MGA) in the adaptation of anticancer treatments and help make treatment decisions in these geriatric patients. The study concluded that the assessment could help gastroenterologists adapt treatment plans for oncological treatment^43^. There are also further methods of frailty assessment such as the Charlson Comorbidity Index and the ‘Multidimensional assessment for cancer in the elderly’ also known as MACE. Through consideration of several factors, these tools can assist in determining prognosis.

Frailty assessment has been readily adopted into professional practice in geriatric medicine. Macmillan Cancer Support, backed by Age UK has collaborated with the department of health to fund further studies on older cancer patients, an important step towards valuable research and making progress^6^. On the basis of the clear advantages of geriatric assessment, funds will also be dedicated towards establishing new means of evaluation to improve selection of cancer treatment. In addition, Macmillan aims to address current issues of ageist prejudice. All these methods of geriatric assessment have resulted in changes in treatment proposals and have been particularly effective in evaluating whether a patient is fit for surgery^22^^,^^41^. Patient health assessment in the geriatric population allows for a comprehensive evaluation of patient health and frailty, helping to overcome the issue of under-treatment due to health perception based on age alone.

Appropriate treatment of geriatric cancer patients can lead to prognostic improvements. Two cohorts of patients aged 70 years or older were compared in a study contrasting the outcomes and effectiveness of oncogeriatric care *vs.* standard care. The cohorts had similar baseline characteristics such as their stage of breast cancer. The results presented how patients receiving standard care obtained fewer modalities of treatment, particularly less surgery, chemotherapy and hormone therapy (*P*<0.001). More importantly, the three-year mortality rate for the group receiving standard care was 71% (95% CI, 61%-83%) in comparison to 58% (95% CI, 42%-75%) for patients in the oncogeriatric care group. This study concluded that more bespoke, cancer-specific care is beneficial in reducing mortality rates and provides an improved prognosis. The study showed how more appropriate care could change survival and emphasised the requirement for further research to quantify the extent of these improvements^44^.

## Future care

Fortunately some progress has been made in the right directions. A number of recent trials by the National Cancer Institute (NCI) have demonstrated the improvement in clinical research regarding oncogeriatric medicine. The institute acknowledges the necessity and clinical requirement for further focused research on geriatric patients^45^. However, barriers still exist for research in geriatric cancer patients. A study published in 2003 by the ASCO investigated the barriers to clinical trial participation by the older women with breast cancer. The study aimed to establish whether the under-representation was due to fewer older women being asked to take part or conversely more of them declining to participate. Seventy-seven pairs were assigned consisting of one older woman aged 65 or above and one younger woman under 65 and these pairs were interviewed about their reasons for participation or refusal. Clinicians offering trials were also questioned about their motives for offering or not offering a trial to an individual. About 68% of younger patients were offered a trial compared to 34% of patients over 65 (*P*=0.004). The most influential reasons for trial offering appeared to be age and stage of cancer. Once offered a trial, there was no significant difference between participation (*P*=0.67), 56% of younger patients accepted and 50% of older patients did the same^46^. The study concluded that the largest obstacle for older women for contributing to a trial was the physicians’ perception about age and tolerance for potential toxicity, both influenced by an ageist approach.

## Conclusion

Current opinions and evidence regarding geriatric oncology and under-treatment all appear to draw similar conclusions. It is universally agreed that there is a lack of evidence for management of elderly cancer patients. Involvement of more elderly patients in clinical trials will allow evidence-based decisions to be made when considering the most effective management for geriatric oncology patients. The risks associated with the treatment options for cancer are present regardless of age; awareness of age is an important factor, but this prejudice needs to be removed. The use of tools enabling geriatric and frailty assessment, such as the CGA, helps to surpass exclusion of treatment due to any age discrimination. Every patient should be educated about all available treatment options to allow them to make an informed decision. In order to make this informed decision and consent, an individual must have capacity. This means they must be able to understand, retain and weigh up information and then communicate a decision^28^. Assumptions should never be made regarding capacity based on age and it should be presumed that every patient has capacity until proven otherwise. Elderly patients should be fully informed about treatment and no information should be withheld or overlooked on the basis of their age. Such presumptions and perceived lack of capacity can lead to under-treatment^19^.

The future of geriatric oncology involves taking a large stride away from the traditional paternalistic approach to treatment, moving to a more patient-centered, tailored approach with well-integrated standardised holistic assessment^2^. Guidelines implemented by institutions such as the SIOG are beginning to recognise the importance of an individualistic methodology not influenced by age. They are endorsing steps towards a more equivalent approach to care. Without such guidelines we are left with a spectrum of care offered which often results in under-treatment of elderly cancer patients and rarely provides the optimal care geriatric patients need.
